# The COVID-19 pandemic: catching up with the cataclysm

**DOI:** 10.12688/f1000research.24963.1

**Published:** 2020-06-23

**Authors:** Surajit Chakraborty, Anirban Basu

**Affiliations:** 1 National Brain Research Centre, Manesar, Gurugram, Haryana, India

**Keywords:** SARS-CoV-2, Public health, Virus, Pandemic, COVID-19

## Abstract

Infection caused by severe acute respiratory syndrome coronavirus 2 (SARS-CoV-2), which belongs to the Coronaviridae family and is a positive-sense single-stranded RNA virus originating from Wuhan, China, was declared a global public health emergency on 11 March 2020. SARS-CoV-2 infection in humans is characterized by symptoms such as fever and dyspnea accompanied by infrequent incidence of lymphopenia, gastrointestinal complications such as elevated hepatic aminotransferases, and diarrhea. Originating in bats, the SARS-CoV-2 virus has been transmitted to humans likely via an intermediate host that is yet to be discovered. Owing to the absence of any vaccines or definite anti-viral drugs alongside the greater mobility of people across the globe, international and national efforts in containing and treating SARS-CoV-2 infection are experiencing severe difficulties. In this review, we have provided a picture of SARS-CoV-2 epidemiological characteristics, the clinical symptoms experienced by patients of varying age groups, the molecular virology of SARS-CoV-2, and the treatment regimens currently employed for fighting SARS-CoV-2 infection as well as their outcomes.

## Introduction

Following the emergence of atypical pneumonia in Guangdong province in 2002–2003, scientific investigators in Hong Kong discovered severe acute respiratory syndrome coronavirus (SARS-CoV) as the causative agent. SARS-CoV spread across the globe, infecting approximately 800 people with a case fatality rate of about 10%. Infection by SARS-CoV was accompanied by symptoms such as fever, coughing, and respiratory distress often leading to pneumonic changes in the lungs ^1^. Around 2011–2012, the world experienced the emergence of another coronavirus, Middle East respiratory syndrome coronavirus (MERS-CoV). Upon infection by the latter, patients demonstrated symptoms and CT findings similar to those seen in the SARS-CoV outbreak ^2^. Although MERS-CoV exhibited a greater case fatality rate than did SARS-CoV, human-to-human transmission was seldom observed. Here, we review the current findings regarding the emergence of the novel coronavirus (SARS-CoV-2) in Wuhan, China, in 2019, its origin, epidemiology, plethora of associated clinical findings, and molecular virology, and the therapeutic strategies being subjected to investigation to combat the global health emergency triggered by this virus.

## Origin and epidemiology

Since 2019, infection by SARS-CoV-2, which belongs to the Coronaviridae family, has resulted in an international global health issue. As of 28 March 2020, according to a World Health Organization report, 512,701 people globally have been infected, resulting in 23,495 deaths. Following the emergence of pneumonia-like cases of unknown origin in Wuhan, Hubei Province, China, deep sequencing analysis from patients’ lower respiratory tract samples revealed the cause of illness as a novel betacoronavirus, 2019-nCoV. Further analysis of genomes sequenced from the bronchoalveolar lavage (BAL) fluid of nine patients revealed that the genome of the causative agent bears about 79% and 50% similarity to SARS-CoV and MERS-CoV, respectively ^3^. On the other hand, maximum sequence similarity was observed with the bat coronaviruses bat-SL-CoVZC45 and bat-CoVZXC21 (87.99% and 87.23%, respectively). The eight complete genomes of 2019-nCoV isolated displayed about 99.98% sequence identity with each other, thus pointing to its very recent emergence in the human population. Work by Yuen and colleagues also demonstrated that 2019-nCoV genomes exhibit greatest similarity with bat SARS-like coronaviruses bat-SL-CoVZC45 and bat-CoVZXC21 ^4^. Although the origin of the 2019-nCoV has been traced to bats, reports point towards the possibility of pangolins as a probable origin for SARS-CoV-2 ^5^. Not only does the pangolin-CoV possess 91.02% similarity with 2019-nCoV at the whole genome level but also five critical amino acids of the S1 domain of 2019-nCoV spike (S) protein were found to be conserved in pangolin-CoV. The authors suggested possible divergence of pangolin SARS-CoV into two distinct lineages, one capable of infecting bats (RaTG13) and the other humans (SARS-CoV-2). Comparison of similarities in nucleotide sequence and S protein receptor-binding domain amongst RaTG13, SARS-CoV-2, and pangolin SARS-CoV led to the formulation of this divergence theory pointing towards the role of pangolin SARS-CoV as the probable closest common ancestor to RaTG13 and SARS-CoV-2. However, the authors did not provide any hint indicating the chance of emergence of SARS-CoV-2 directly from pangolin SARS-CoV-2. A study by Liu *et al*. ^6^, however, pointed directly to the fact that SARS-CoV-2 did not evolve directly from pangolin SARS-CoV on the basis of S-gene nucleotide sequence comparison amongst bat coronavirus, pangolin SARS-CoV, and SARS-CoV-2. The authors also support the view that SARS-CoV-2 originated via multiple recombination events in nature whose exact sequence determination will necessitate further investigations. In addition to the theory that natural selection acted upon 2019-nCoV in an animal host prior to zoonotic transfer, the possibility of natural selection in humans post-zoonotic transfer has been opined by some ^7^. It might also be possible that the SARS-CoV-2 progenitor, while undergoing transmission in humans following zoonotic transfer, acquired genomic features necessary for the pandemic to take off.

Li *et al.* ^8^ describe the epidemiological characteristics of nCoV-induced pneumonia (NCIP) by studying the demographic information, exposure history, and illness timelines of confirmed cases of SARS-CoV-2 reported by 22 January 2020. In brief, the median age of patients confirmed with NCIP was 59 years. A total of 55% of the NCIP cases reported before 1 January 2020 were found to be directly associated with a visit to the Huanan seafood wholesale market in contrast to very few (8.6%) of the subsequent cases, thus providing evidence of human-to-human transmission occurring among close contacts. The mean incubation period was found to be 5.2 days, with the 95 ^th^ percentile of distribution at 12.5 days. During this early period of outbreak, using the mean serial interval, which was calculated to be 7.5 days, the reproductive number was estimated to be 2.2. The SARS-CoV-2 outbreak, with its epicenter in Wuhan, was demonstrated to result in a possible nosocomial spread and beyond Wuhan ^4^. Patients, after contracting the virus in Wuhan, were found to travel to Shenzhen, thus infecting those who did not have any history of travelling to Wuhan. This study was also the first to identify an asymptomatic male patient (10 years old) whose lung CT scan presented with “ground-glass opacity” pneumonic changes. Subsequent to the spread of the virus within Wuhan and outside, reports began to emerge suggesting the spread of the virus beyond Chinese national boundaries. Observations published suggested the spread of the SARS-CoV-2 virus outside China to Taiwan ^9^ and the United States ^10^. Another study by Sun *et al.* ^11^ reported the epidemiological profile of the SARS-CoV-2 infection of 507 patients from mainland China as well as exported cases on the basis of a crowdsource platform (from 13 to 31 January 2020). The age distribution of SARS-CoV-2 patients displayed a skew towards older age groups, with a median age of 45 years. The median age of the patients who had died during reporting was observed to be 70 years, thus indicating that older age groups are at greater risk of poor outcomes. The relative risk measurement for the age group below 15 years was found to be significantly low, i.e. 0.5. A report by Wu *et al.* ^12^ provided an estimation of the clinical severity of SARS-CoV-2 infection based upon data up to 28 February 2020. The authors used a transmission dynamics model updated with real-time input data and data from additional sources to predict symptomatic case fatality risk (sCFR), the relative susceptibility to symptomatic infection on the basis of different age groups. Overall, the sCFR in Wuhan was found to be 1.4% (0.9–2.1%). When the sCFR was analyzed on the basis of different age groups, it was found to be 0.3%, 0.5%, and 2.6% for <30 years, 30–59 years, and >59 years, respectively. The age groups <30 years and >59 years were found to be 0.16 and 2.0 times more susceptible to symptomatic infections, respectively. In addition to these clinical severity measures, the basic reproduction number and mean serial interval were observed to be 1.94 (1.82–2.06) and 7.0 (5.8–8.1) days, respectively. However, it should be kept in mind that the basic reproductive number varies widely depending upon socio-economic, behavioral, biological, and environmental factors and hence must be interpreted with deep caution ^13^.

## Molecular virology: genome characteristics, spike protein structure, and its implication in human ACE2 receptor binding

Coronaviruses comprise a group of viruses whose infection is characterized by signs and symptoms such as fever, respiratory distress, gastrointestinal complications, etc. The genomes of coronaviruses range from 26 to 32 Kb and consist of 6 to 11 open reading frames (ORFs) ^14^. The first ORF, which comprises up to nearly 67% of the entire genome, codes for 16 non-structural proteins (nsps) ^15^. On the other hand, the remaining ORFs code for accessory and four structural proteins, namely matrix (M), S, envelope (E), and nucleocapsid (N) proteins. A study published by Lu *et al.* ^3^ provided one of the first reports stating the characteristics of the SARS-CoV-2 genome and the putative human receptor utilized for its entry. Whole genome sequencing followed by comparison revealed 87.99% and 87.23% sequence similarity with SARS-like bat coronaviruses bat-SL-CoVZC45 and bat-SL-CoVZXC21, respectively, which was found to be significantly greater than its similarity with SARS-CoV and MERS-CoV (79% and 50%, respectively). Comparison of the predicted coding regions of 2019-nCoV with bat-SL-CoVZC45 and bat-SL-CoVZXC21 revealed similar genome organization and 12 predicted coding regions (1ab, S, 3, E, M, 7, 8, 9, 10b, N, 13, and 14). The lengths of most proteins encoded by 2019-nCoV were found to be similar to those of bat SARS-like coronaviruses, with only a few minor modifications. The S protein of 2019-nCoV was found to be longer than that of bat SARS-like coronaviruses, SARS-CoV, and MERS-CoV. Although the 2019-nCov S protein possesses 76.2% sequence similarity with that of SARS-CoV, S1 domain of 2019-nCoV possesses 50 amino acids which were found to be conserved in S1 domain of SARS-CoV. Even gene sequence comparison revealed a greater similarity between 2019-nCoV receptor binding domain of S protein and SARS-CoV than bat-SARS-like coronaviruses. Analysis of the 2019-nCoV genome by another group ^16^ suggested 14 ORFs encoding for 27 proteins. Genes comprising ORF1ab and 1a were observed to be located at the 5' terminus, thus encoding pp1ab and pp1a proteins, respectively. Together, pp1ab and pp1a encode 15 nsps including nsp1 to nsp10 and nsp12 through nsp16. The 3' terminus was found to harbor genes for S, E, M, N, and eight accessory proteins (3a, 3b, p6, 7a, 7b, 8b, 9b, and orf14). As documented by Lu *et al.*, the protein sequence of 2019-nCoV was found to be similar when compared to SARS-CoV ^16^, although certain differences were observed and deserve special mention. The accessory protein 8a found in SARS-CoV was not present in 2019-nCov. The 8b protein of 2019-nCov was found to be larger (124 amino acids) than that of SARS-CoV (84 amino acids). On the contrary, 2019-nCoV protein 3b was discovered to significantly differ in length (22 amino acids) to that of SARS-CoV (154 amino acids). Combined, 380 amino acid substitutions were found in 2019-nCoV and the respective protein sequences of SARS-CoV and bat-SARS-like coronaviruses. However, no amino acid sequence differences were observed for proteins like nsp7, nsp13, E, M, p6, and 8B. Further research investigating the significance of the differences in amino acid sequence thus might provide insight into the differences in host selection, transmissibility, tropism, and pathogenicity.

Coronaviruses use S protein for entry to target cells. S protein consists of two functional subunits, S1 and S2. The S1 subunit contributes to receptor binding followed by the fusion of host cell endosomal membrane with viral envelope aided by S2 subunit. Following interaction of the S1 subunit with the cellular receptor, host-derived proteases are known to cleave at the S1/S2 and S2' junctions, resulting in a series of irreversible conformational changes, hence culminating into nucleocapsid entry ^17– 19^. One of the notable features of the 2019-nCoV S protein is a polybasic Q _677_TNSPRRARSV _687_ site situated at the S1/S2 junction ^7^. Numerous such polybasic sites were found in the S protein sequence of multiple highly pathogenic avian influenza viruses ^20^, which upon abrogation culminated in the amelioration of their pathogenicity. However, upon mutation of the furin-mediated cleavage site within the aforementioned sequence, SARS-CoV-2-mediated entry into Vero and human angiotensin-converting enzyme 2 (hACE2)-expressing cells was moderately affected, hence indicating its possible role in virus tropism and transmissibility. As suggested by Andersen *et al.* ^7^, the incorporation of proline into the polybasic site might result in the addition of O-linked glycans flanking the cleavage site, thus helping to form mucin-like domains, which in turn can shield epitopes or key residues in the 2019-nCoV S protein ^21^.

Phylogenetic analysis of the receptor-binding domain of four different lineages of coronaviruses revealed a closer relationship between 2019-nCoV and SARS-CoV than with bat-SL-CoVZC45 and bat-SL-CoVZXC21, despite high similarity of 2019-nCoV with the latter two at the whole genomic level ^3^. In addition, since 76% similarity was obtained at the level of amino acid sequence of S protein of 2019-nCoV and SARS-CoV, the role of the hACE2 protein (which was reported to act as the receptor for SARS-CoV) as a putative receptor for 2019-nCoV was subjected to intensive analysis. A study by Zhou *et al.* ^22^ provided the first evidence for the role of ACE2 as the cellular entry receptor for 2019-nCoV. Infection of HeLa cells expressing hACE2 protein resulted in infection by 2019-nCoV, which was found to be reversed in the absence of hACE2, thus proving that hACE2 indeed plays a role in the entry of 2019-nCoV. Moreover, this study also showed that 2019-nCoV does not use other coronavirus receptors like aminopeptidase N (APN) and dipeptidyl peptidase 4 (DPP4) for its entry into host cells. In agreement with previous findings, a report by Hoffmann *et al.* ^23^ also supported the role of hACE2 as the entry receptor protein for 2019-nCoV. Directed expression of hACE2 protein or bat ACE2 resulted in infection of non-susceptible BHK-21 cells by 2019-nCoV. In addition to the role of human ACE2 protein in viral entry, the report also mentioned the role of endosomal proteases cathepsins B and L and serine protease TMPRSS2 in the priming of S protein for 2019-nCoV entry. Ammonium chloride-mediated failure in acidifying the endosomes resulted in inhibition of SARS-CoV-2 S-mediated viral entry, pointing towards a role for cathepsins B and L. The clinically proven TMPRSS2 inhibitor camostat mesylate was also demonstrated to inhibit viral entry mediated by 2019-nCoV S protein. Full inhibition of S protein-induced viral entry was reported when camostat mesylate and E-64d and an inhibitor of cathepsin B/L were added together, thus revealing a cohesive role for TMPRSS2 and cathepsin B/L in S protein priming and viral entry.

## Spectra of clinical characteristics in patients suffering from 2019-nCoV infection

**Figure 1.**
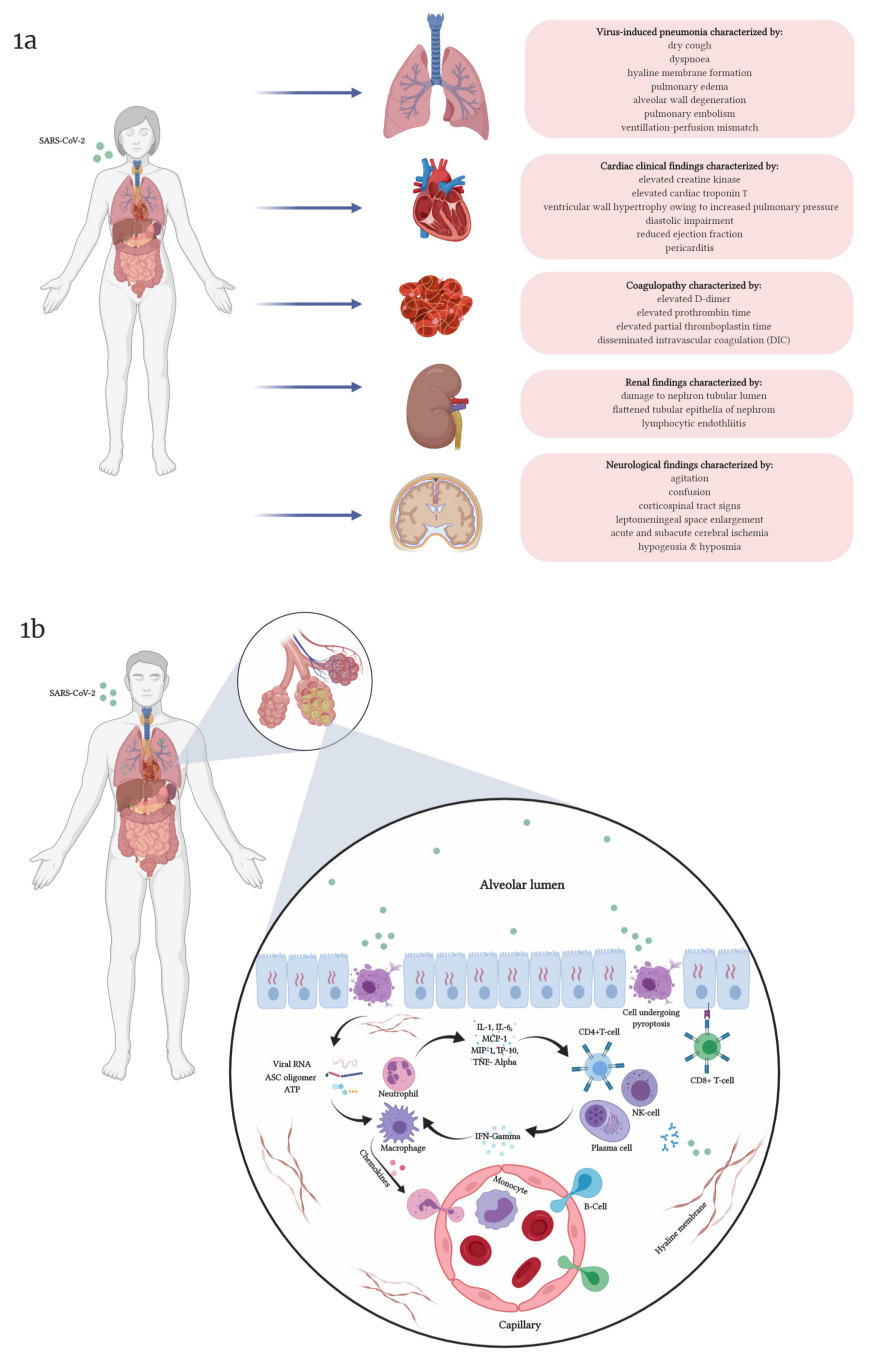
Severe acute respiratory syndrome coronavirus (SARS-CoV)-2-induced clinical symptoms and the mechanistic basis culminating in infection-induced cytokine storm. Figure 1a depicts the major organ systems affected in the course of SARS-CoV-2 infection in patients. The involvement of pulmonary, cardiovascular, renal, and nervous systems observed in patients has been shown in the figure with major clinical findings related to respective organ dysfunction. Figure 1b elucidates the mechanistic basis for pulmonary immunopathology associated with SARS-CoV-2 infection. Owing to infection of alveolar pneumocytes by SARS-CoV-2, cells undergo pyroptosis, upon which chemicals like ATP molecules, ASC oligomers, and viral RNA are released. These chemicals play a major role in activating alveolar macrophages. Alveolar macrophages, upon activation, result in the synthesis and secretion of a battery of chemokines and cytokines into the interstitial space. Chemokines promote the extravasation of granulocytes and lymphocytes into the lung parenchyma to help combat the infection. Macrophages and neutrophils secrete proinflammatory mediators such as interleukin (IL)-1, IL-6, monocyte chemoattractant protein (MCP)-1, macrophage inflammatory protein (MIP)-1, interferon gamma (IFN-γ)-induced protein 10 (IP-10), and tumor necrosis factor (TNF)-α, thus activating adaptive immune cells (such as T-helper cells, etc.) and natural killer (NK) cells to secrete IFN-γ, which in turn stimulates neutrophils and macrophages to secrete IL-1, IL-6, MCP-1, MIP-1, IP-10, and TNF-α. This vicious cycle of inflammation culminates in unwanted tissue damage, resulting in the pulmonary pathology observed in COVID-19.

The battery of clinical manifestations upon infection by SARS-CoV-2 infection ranges from common symptoms like fever, cough, and myalgia to infrequently reported rhinorrhea, confusion, dizziness, vomiting, and diarrhea ( Figure 1a). A study published by Huang *et al.* ^24^ reported that most patients present with fever, dry cough, dyspnea, and ground-glass opacity on chest CT scans, resembling the clinical features documented in SARS-CoV and MERS-CoV infections ^2, 25^. Very few patients also presented with the same for upper respiratory tract infections like sneezing, sore throat, and rhinorrhea. However, less common symptoms like headache, hemoptysis, and diarrhea were also observed in a few of the patients from the cohort. Unlike SARS-CoV and MERS-CoV infections presenting with gastrointestinal complications like diarrhea, vomiting in 20–25% of cases ^2, 25^, the aforementioned study also demonstrated that only 8% of the cohort experienced diarrhea. Biochemical analysis demonstrated increased plasma IL1B, IL1RA, IL-7, IL-8, IL-9, IL-10, basic FGF, GCSF, GMCSF, interferon (IFN)-γ, IP-10, MCP-1, MIP-1A, MIP-1B, PDGF, TNF-α, and VEGF concentrations compared to healthy adults. In addition, ICU-admitted patients exhibited higher plasma abundance of IL-2, IL-7, IL-10, GCSG, IP-10, MCP-1, and TNF-α than did non-ICU-admitted patients. Thus, 2019-nCOV patients were shown to demonstrate activation of both Th1 and Th2 arms of immunity, which differs from other SARS-CoV infections ^26^. In agreement with the previous report, Chen *et al.* ^27^ also reported a greater prevalence of common signs and symptoms like fever, dry cough, and dyspnea compared to less common symptoms like muscle ache, confusion, headache, nausea, vomiting, and diarrhea. Elevated serum alanine aminotransferase, aspartate aminotransferase, lactate dehydrogenase, and lymphopenia were observed in a substantial number of patients, as shown by Huang *et al.* ^24^. Although elevated hepatic aminotransferases in the blood of 2019-nCoV patients may indicate acute liver injury via viral hepatitis, Bangash *et al.* ^28^ suggested that the elevation of aminotransferase concentration may arise as a result of altered immune interactions involving hepatic cytotoxic T-cells and Kupffer cells. A report explaining radiological findings from 2019-nCoV patients ^29^ provides a detailed account of pulmonary architecture upon 2019-nCoV infection. The mean number of lung segments involved was observed to be 10.5. A predilection was observed for right lower lobes, which is probably attributed to the short and straight anatomical structure of the right bronchus. CT scans of patients with subclinical illness exhibited unilateral involvement with multifocal ground-glass opacities. During the first week of symptom appearance, bilateral involvement was noticed. The lesion was diffuse in nature with a reduction in the frequency of ground-glass opacity, followed by transition to consolidation and mixed-pattern development during the second week post-symptom onset. Interlobular or interseptal thickening was noted, suggesting a resemblance to CT findings from SARS-CoV ^30, 31^ and MERS-CoV infections ^32, 33^. The authors also proposed a positive correlation between NCIP and old age as well as comorbidities such as chronic obstructive pulmonary disease, diabetes, and other chronic pathological states. Multiple reports highlighting the cardiac perturbations observed in the context of SARS-CoV-2 infection are currently emerging. A study by Inciardi *et al.* ^34^ documented a case study of a 53-year-old female patient where SARS-CoV-2 infection was observed to be associated with multiple cardiac complications. The patient’s left ventricular wall exhibited an increase in thickness, diastolic impairment, reduced ejection fraction, and circumferential pericarditis. Another report by Creel-Bulos *et al.* ^35^ reported incidences of acute cor pulmonale in patients suffering from SARS-CoV-2 infection. The complication was associated with right ventricular dilatation with systolic impairment and presence of pulmonary thromboembolism. Although SARS-CoV-2 has been hypothesized to be able to infect cardiomyocytes owing to their high expression of ACE2 and incidences of coagulopathy in COVID-19, further studies are necessary before concluding that there is any causative relationship between SARS-CoV-2 infection and cardiological malfunctions.

Helms *et al*. ^36^ reported neurological features of 58 patients suffering from COVID-19. A total of 84% of the patients exhibited neurological features like agitation and confusion, corticospinal tract signs (hyperreflexia), leptomeningeal space enlargement, and bilateral frontotemporal hypoperfusion. However, not all signs and symptoms were being noticed together in any particular individual patients. Few patients upon magnetic resonance imaging (MRI) were observed to have experienced acute/sub-acute ischemic stroke. A similar report by Mao *et al.* ^37^ ** documented a case series and the neurological features associated with SARS-CoV-2 infection based upon patients’ subjective descriptions. Neurologic complications including cerebrovascular disease, conscious disturbance, and skeletal muscle injury were observed in 36.4% of patients. The prevalence of taste and smell disorders was also observed in a minority (5%) of patients. Incidences of loss of smell and/or taste were also demonstrated by Bénézit *et al.* ^38^ in a retrospective study of data collected using a web-based questionnaire. Hypogeusia and hyposmia were found to be strongly correlated with COVID-19 both separately and in a combined fashion. Since the nervous system possesses ACE2 receptors, exposure of the olfactory epithelium to the virus may help contribute to the manifestations described above. In agreement with the notion that SARS-CoV-2 possesses neuroinvasive potential, a case study reported by Moriguchi *et al.* ^39^ described the neurological findings of a 24-year-old male patient upon infection by SARS-CoV-2. Viral RNA was detected in the cerebrospinal fluid (CSF). The patient was found in an unconscious state and was observed to experience transient generalized seizure during transit to the hospital. Imaging resulted in the discovery of right lateral ventriculitis, right medial temporal lobe encephalitis, and hippocampal atrophy. Further reports mentioning neurological symptoms and imaging findings will help understand the dynamic spectra of manifestations. Investigations using animal models to elucidate neuroinvasive mechanisms will further help unfold SARS-CoV-2 biology, thus providing significant insights for the development of future therapeutic strategies.

In accordance with the previous study, results stated by Yang *et al.* ^40^ similarly showed a positive correlation among old age, comorbidities like diabetes, and 2019-nCoV infection-induced death. Out of the 52 critically ill patients enrolled for the study (mean age: 59.7 years), 32 patients died, resulting in a mortality rate of 61.5%. The mean age of survivors was also found to be less when compared to that of non-survivors (51.9 years versus 64.6, respectively), thus indicating a poor outcome of infection in critically ill patients with greater age. Analysis of upper respiratory specimens of infected patients (age ranging from 26–76 years) revealed higher viral loads with onset of symptoms followed by a gradual decline with the progression of time ^41^. Moreover, viral load found in the nasal passageway was greater than in the throat, which resembles that of influenza ^42^ but not of SARS-CoV infection ^43^. Besides the prevalence of co-morbid conditions (such as diabetes mellitus and hypertension), the other determining factors deciding increased mortality of elderly COVID-19 patients are still under investigation. However, altered pulmonary dendritic cell maturation and T-cell activation might contribute to amplified immune reaction in the lungs, resulting in increased mortality ^44^. Based upon the future demographic scenario according to the World Health Organization, which predicts the population size above the age of 65 years to be almost double that under 5 years of age, the effect of SARS-CoV-2 infection on elderly patients underscores the importance of elucidating the mechanistic differences in the immune response and thus pathology in elderly and non-elderly patient groups ^45^.

Lu *et al.* ^46^ provide evidence for infection in children being accompanied by a milder clinical course when compared to that in adults. Lymphopenia was observed in 3.5% of children (6 out of 171). In addition to fever, the other common signs and symptoms included cough and pharyngeal erythema. A similar study by Liu *et al.* ^47^ also points towards lymphopenia observed in children infected with 2019-nCoV. Lesser severity of clinical symptoms in pediatric patients when compared to adults has also been demonstrated by Xu *et al.* ^48^. Although fewer cases of lymphopenia, leukopenia, or elevated transaminases were observed than in adults, rectal swabs of 8 out of 10 patients were found to be positive for viral RNA as quantified by real-time RT-PCR. Shedding of viral RNA was found to continue even after viral RNA became undetectable in nasopharyngeal swabs. Qui *et al*. ^49^ published a study in concordance with the aforementioned ones, demonstrating less severe signs, symptoms, and laboratory findings in pediatric patients with a mean age of 8.3 years. A large proportion of asymptomatic patients were observed, thus highlighting the implication of the latter upon controlling disease spread. However, multiple recent reports of pediatric COVID-19 cases point towards variable disease severity, signs, and symptoms. A report by Pain *et al.* ^50^ documented an atypical case of a 14-year-old COVID-19 pediatric patient presenting without respiratory distress. Gradually, the patient developed symptoms like lymphopenia, increased D-dimer, elevation of serum ALT and AST, dyspnea, and cytokine storm. Upon treatment with the IL-1 inhibitor anakinra, conditions were found to improve followed by the absence of viral RNA in nasopharyngeal samples and stool. Similar findings were reported by Dallan *et al.* ^51^, where adolescent patients with an age ranging from 10–12 years were found to present with hypotensive shock, compensated shock with cold periphery and late capillary filling time, and multi-organ dysfunction syndrome (MODS). Transmission of SARS-CoV-2 in premature infants has also been documented to be associated with elevation of serum inflammatory markers, respiratory distress, and MODS (associated with renal, liver, and bone marrow dysfunction) ^52^. Administration of the IL-6 inhibitor tocilizumab and remdesivir eventually resulted in the restoration of respiratory physiology and improvement of organ functions. These studies thus highlight the variability of signs and symptoms presented by pediatric COVID-19 patients and its significance in the early and proper diagnosis of these patients in order to avoid poor disease outcomes.

Data published by Chen *et al.* ^53^ evaluated the clinical characteristics and risk for intrauterine vertical transmission of SARS-CoV-2 in pregnant women undergoing Cesarean section. No differences in clinical characteristics were observed in pregnant patients with respect to non-pregnant adults. Chest CT scans revealed multifocal bilateral ground-glass opacity. Common symptoms exhibited by patients consisted of fever and cough, whereas the less common ones were sore throat, myalgia, malaise, diarrhea, and shortness of breath. Similar evidence was also provided by Li *et al.* ^54^, stating that clinical manifestations of pregnant women upon 2019-nCoV infection did not differ from those of non-pregnant adults. Chen *et al*. ^53^ showed no vertical transmission of virus, as demonstrated by the absence of viral RNA in amniotic fluid, cord blood, and neonatal throat swab. Although one out of seven neonates tested positive for viral RNA on throat swab 36 hours after birth ^54^, the placenta and cord blood were found to be negative for 2019-nCoV RNA, thus warranting further investigation to determine intrauterine vertical transfer.

## General pathology and immunopathogenesis

Multiple autopsy reports of patients suffering from COVID-19 have surfaced, providing a vivid description of the pathology associated with infection with SARS-CoV-2 ^55– 57^. Reported findings of autopsy examination of deceased COVID-19 patients revealed incidences of pulmonary embolism and reticular infiltration of lungs with bilateral diffuse ground-glass opacity on CT scans. Pulmonary interstitium appeared to be heavy and firm upon gross examination. Examination of cut surfaces of lung tissue exhibited alternate areas of tan-gray consolidation with patchy hemorrhagic areas. Histological analyses of pulmonary tissue indicated activated pneumocytes, microvascular thrombosis, capillary congestion due to deposition of fibrin thrombi, alveolar basement membrane edema, alveolar septum degeneration, and hyaline membrane deposition, thus culminating in ventilation-perfusion mismatch. In addition, both granulocytic and lymphocytic infiltration was observed in the pulmonary interstitium. Incidences of deep vein thrombosis were also noted in a significant proportion of patients. The presence of CD61 ^+^ megakaryocytes with nuclear hyperchromasia and atypia were also denoted by autopsy findings. Entrapment of neutrophils in fibers deposited in the pulmonary interstitium indicate a role for neutrophil extracellular traps (NETs) in COVID-19 pathogenesis.

Cardiac findings like myocardial hypertrophy and focal cardiomyocyte necrosis were found ^56, 57^ upon autopsy studies. Although cardiac muscle fibers were subjected to necrotic death, rare infiltration of lymphocytes adjacent to (but not surrounding) the necrotic cells rules out the development of virus-induced myocarditis. The abovementioned studies were successful in isolating viral genomic RNA from the liver, kidney, and brain, denoting viral spread from pulmonary to distant tissues. Analysis of renal tissue revealed damage to the nephronal tubular lumen and epithelium along with virus-like structures in podocytes and glomerular endothelial cells. A study by Varga *et al.* ^58^ indicated lymphocytic endotheliitis in the vasculature of organs such as the heart, lungs, liver, small intestine, and kidneys upon post-mortem analysis, thus underscoring the importance of vascular physiology in maintaining hemostasis and preventing multi-organ dysfunction.

The pulmonary pathology observed in the course of COVID-19 progression has been generally attributed to direct effect due to viral propagation and the exaggerated wave of inflammatory response imposed to combat viral infection ( Figure 1b). Infection of pneumocytes by SARS-CoV-2 has been reported to result in upregulation of hACE2 receptors, thus further enhancing viral entry into the cell and promoting viral propagation ^59^, which ultimately leads to pneumocyte death and pulmonary pathology. This virus-induced ACE2 upregulation has been observed to be mediated in an IFN-dependent fashion. On the other hand, one report ^24^ indicates a role for an exaggerated inflammatory response as exemplified by the upregulation of serum proinflammatory markers such as IL-6, IP-10, MCP-1, and MIP-1α upon severity of COVID-19, thus indicating the induction of a severe cascade of proinflammatory reactions and its effect upon disease pathogenesis. In addition, serum level D-dimer was observed to be enhanced in critical COVID-19 patients, suggesting induction of coagulopathy. Besides the insight provided by autopsy studies regarding the immune landscape of the pulmonary system in the context of COVID-19, work by Liao *et al.* ^60^ demonstrated the role of FCN1 ^+^ macrophages in aggravating the severity of pulmonary pathology upon infection by SARS-CoV-2. FCN1 ^+^ macrophages are characterized by high levels of chemokine production implicated in the cytokine storm. Indeed, an increased population of FCN1 ^+^ macrophages was found in the BAL fluid from critically ill COVID-19 cases when compared to that from mild cases, thus implicating its role in resulting overt inflammatory sequence of events followed by poor prognosis. Pneumocyte pyroptosis upon infection by SARS-CoV-2 results in the secretion of a plethora of immune-activating molecules. These molecular species typically exhibit immunostimulatory effects upon the innate immune cells of the lungs, including alveolar macrophages, thus eliciting a wave of inflammatory reactions. The cytokines and chemokines secreted by these innate immune cells further help in the recruitment of adaptive immune cells like CD8 ^+^ T-cells, CD4 ^+^ T-cells, and B-cells ^61^. Extravasation of these adaptive immune cells with NK cells upon the call emanating from inflamed pulmonary interstitium leads to activation of these newly secreting cells, which further respond by secreting proinflammatory mediators such as IFN-γ. These chemicals help in further activation of already activated innate immune cells, hence creating a vicious inflammatory reaction. Indeed, reduced type I and III IFN abundance in blood transcriptome accompanied by enhanced cytokine (including IL-6) and chemokine levels are speculated to contribute to COVID-19 pathogenesis ^62^. The lymphopenia in serum accompanied by SARS-CoV-2 infection is hypothesized to result from this altered distribution of lymphocytes from serum to lung parenchyma, as suggested by Cao *et al.* ^63^. However, some results ^64^ point towards the role for direction infection-induced exhaustion of CD8 ^+^ T-cells, thus resulting in the diminution of blood lymphocyte count. SARS-CoV-2-induced lymphopenia along with elevated neutrophil count in blood comprise the increased neutrophil-to-lymphocyte ratio, which has been implicated as an independent risk factor negatively regulating disease outcome ^65^. This elevated neutrophil-to-lymphocyte ratio has been observed to be associated with NET formation in serum ^66^. Exposure of *in vitro* neutrophils from healthy control subjects to COVID-19-derived patient serum induced NET formation, thus suggesting a role for an as-yet-unknown factor promoting the latter. Moreover, the propensity of NET formation was indeed found to be positively correlated with the severity of disease. SARS-CoV-2-induced cytokine storm has also been stated to have implications in the management of patients suffering from systemic lupus erythematous (SLE) ^67^. Owing to higher susceptibility of ROS-induced (for example, in the instance of viral infection) DNA hypomethylation, SLE patients are more prone to ACE2, proinflammatory cytokines, and their transcription factors like NF-κB overexpression, ultimately culminating in enhanced viral propagation and cytokine storm development. Thus, besides having implications in disease progression and outcome, COVID-19-associated cytokine storm warrants strict management of lupus erythematosus in SLE patients in order to avoid overt outcomes in the face of SARS-CoV-2 infection.

Although SARS-CoV-2 infection has been associated with diffuse bilateral alveolar damage, autopsy reports point towards frequent incidences of capillary thrombus and intravascular clotting in both lungs and extra-pulmonary sites. Overt immune response against SARS-CoV-2 has been mentioned to contribute to this diffuse coagulopathy ^63, 68^. The cytokine IL-6 has been implicated in triggering the coagulation cascade activity concomitantly with reduced fibrinolysis. However, owing to high expression of ACE2 receptor in blood vessel endothelial cells, direct cell death as a result of endothelial cell infection by SARS-CoV-2 may predispose the coagulation system to overt activation, hence leading to intravascular clotting disorder. Ventilation-perfusion mismatch resulting in hypoxia may also activate the clotting cascade in the pulmonary vasculature in an inflammation-dependent manner. Further investigations aimed at elucidating the mechanistic basis for this exaggerated immune system activation upon infection by SARS-CoV-2 might provide sufficient insights into the development of therapeutic strategies against COVID-19.

## Therapy and vaccine development: where are we?

Although no preventive or therapeutic strategies against 2019-nCoV exist to date, researchers across the globe are continuing to search for one ( Table 1). In the confrontation of a global health emergency, the repurposing of drugs has become a very popular choice for researchers in the quest for therapeutics.

A report by Zhou *et al.* ^80^ presented an integrative antiviral drug repurposing methodology by employing a system pharmacology-based network medicine platform, which helped to identify the possible therapeutic efficacy of 16 candidate repurposable drugs and three potential drug combinations. Drugs like toremifene, equilin, irbesartan, sirolimus, mercaptopurine, melatonin, and eplerenone, on the basis of their interactions with host proteins and the proximity measures of those target proteins with coronavirus-induced proteome changes, are possible future therapeutics in combating 2019-nCoV infection following further preclinical and clinical studies.

**Table 1.** Summary of the major therapeutic strategies under clinical trial, their respective preliminary outcomes, and the putative mechanisms behind their actions.

Therapy: drug/drug combinations & others	Putative mechanism of action	Recent developments in clinical trials	References(s)
Remdesivir	Possibly by inducing mutagenesis and/or premature viral replication termination	A numerical reduction in time to clinical improvement,albeit not statistically significant	69
Hydroxychloroquine	Interfere with viral entry as well as post-entry steps	Open-label randomized controlled trial indicated no improvement of COVID-19 patients	70
Ritonavir-Iopinavir	HIV-protease inhibitor	No sifnificant reduction in mortality upon drug administration with respect control	71
Favipiravir	Upon incorporation into viral RNA genome, favipiravir helps inhibit RNA-dependent RNA polymerase	Significant improvement in chest CT findings and reduction in virus clearance time	72
Tocilizumab	Monoclonal antibody directed against IL-6, thus hypothesized to ameliorate the intensity of cytokine storm	Drastic improvement in chest CT findings and reduction in oxygen support and serum proinflammatory markers	73, 74
Anakinra	Blocking IL-1α and IL-1β receptor, thus stated to reduce severity of cytokine storm	Prominent improvement in respiratory functions and decline in mortality	75
Interferon beta-1β, Ritonavir-lopinavir, ribavirn	Intereferon beta-1β: by inductiog antiviral immunity Ritonavir-Iopinavir: HIV-protease inhibitor Ribavirin: guanosine analog hypothesized to inhibit viral replication by incorporating itself into viral genome and thus introducing mutations	Significantly rapid virus clearance from nasopharyngeal and oropharyngeal swab and rapid improvement in clinical symptoms	76
Baricitinib	Inhibitor of Janus kinase activation, thus reducing severity of cytokine storm	Prominent improvement in respiratory and clinical functions and significant reduction in time to recovery	77
Convalescent plasma (CP) therapy	Neutralization of SARS-CoV-2 viral particles, thus imparting protection	Improvement in chest radiological findings, clearance of vira RNA, restoration of normal CRP level, and correction of lymphopenia	78
Adn5-nCoV vaccine candidate	Vaccine comprises adenovirus type-5 vector expressing S protein, thus conferring both B- and T-cell immunity	Significant B- and T-cell immunity was observed to be developed upon administration of vaccine	79

Data published by Wang *et al.* ^81^ reported the *in vitro* efficacy of remdesivir and chloroquine in abrogating infection by 2019-nCoV. Remdesivir is a nucleoside analog that has been reported to affect viral propagation either by inducing mutagenesis or by resulting in premature replication termination ^82^. Chloroquine, on the other hand, is an anti-malarial drug that has been shown to possess broad-spectrum anti-viral activity ^83^. Chloroquine has been demonstrated to ameliorate viral entry by inhibiting viral envelope fusion with the endosomal membrane as well by altering the glycosylation status of SARS-CoV receptors ^84^. A report by Wang *et al.* ^81^ illustrated the effects of remdesivir at a post-entry step, whereas chloroquine was observed to inhibit viral propagation at both entry and post-entry stages. A similar study by Liu *et al.* ^85^ assessed the therapeutic efficiency of hydroxychloroquine (HQ) against SARS-CoV-2 infection *in vitro* in comparison to that of chloroquine owing to the fact that HQ is 40% less toxic than chloroquine in animals. HQ was found to inhibit viral propagation at both entry and post-entry steps, albeit with a moderately lower potency than chloroquine. In addition to its antiviral activity, the authors suggest that the anti-inflammatory properties of HQ might also lead to reduced magnitude of inflammatory reaction in the face of cytokine storm elicited by 2019-nCoV, thus reducing inflammation-induced pulmonary damage. The results of an open-label non-randomized clinical trial published by Guatret *et al.* ^86^ indicate improved outcomes in 2019-CoV patients upon administration of HQ with or without azithromycin. In a group of 26 patients, 70% of patients receiving HQ treatment were found to be negative for viral RNA at day 6 post-inclusion. Within the same period of time, 100% of patients receiving HQ along with azithromycin were observed to be negative for viral RNA on nasopharyngeal swab. Contrary to the beneficial effects of HQ shown by the abovementioned trial, a recent multi-center, open-label randomized controlled trial involving 150 participants indicated no improvement in virus clearance upon administration of HQ at a dose of 1,200 mg daily for 3 days followed by a maintenance dose of 800 mg for 2–3 weeks for patients with mild-to-moderate/severe disease, respectively ^70^. In addition, a significantly greater proportion of patients exhibited adverse effects such as diarrhea upon treatment with HQ when compared to standard care, thus raising questions about the real efficacy and safety profile in COVID-19 patients. Similarly, in contrast to the promising outcome of remdesivir action *in vitro* against SARS-CoV-2 infection, a recently published report ^69^ indicated no statistically significant clinical benefits of remdesivir administration in the context of COVID-19. However, a numerical reduction in time to clinical improvement was noted in patients treated early with the drug. Future trials involving a larger number of participants are thus warranted to confirm this finding.

A clinical trial to assess the therapeutic activity of ritonavir-lopinavir combination conducted by Cao *et al.* ^71^ did not show any beneficial effect of therapy in the setting of 2019-nCoV infection. Ritonavir and lopinavir are HIV protease inhibitors which have already been demonstrated to be effective in treating SARS-CoV infection in patients ^87^. The mortality rate was found to be similar in the drug-treated and control group of patients (receiving standard care) at 28 days post-onset of therapy. However, a post-hoc subgroup analysis revealed reduced mortality of patients receiving ritonavir-lopinavir within 12 days of symptom onset when compared to that of patients receiving standard care within the same time window.

Favipiravir is a purine nucleoside analog which, upon entry into the cell, undergoes ribosylation and phosphorylation followed by incorporation into viral RNA genome during virus replication ^88^. Upon incorporation into the viral genome, it helps inhibit viral RNA-dependent RNA polymerase (RdRp) enzyme, thus culminating in abrogated viral replication. Owing to the fact that SARS-CoV-2 is an RNA virus whose replication is dependent upon RdRp, favipiravir was subjected to investigation to assess its therapeutic potential ^72^. Treatment with favipiravir was associated with shorter virus clearance time and significant improvement in chest CT findings, thus indicating its possible use as a treatment strategy for COVID-19. However, a future multi-center controlled randomized trial is necessary before commenting on the efficacy of favipiravir against SARS-CoV-2 infection.

As mentioned previously, the pathogenesis of COVID-19 is believed to heavily depend upon the ensuing cytokine storm against virus propagation. IL-6, being implicated in the SARS-CoV-2-induced cytokine storm, has been targeted using the monoclonal antibody tocilizumab. A report by Xu *et al.* ^73^ demonstrated the outcome of tocilizumab treatment via an intravenous route upon COVID-19 outcome in critically ill patients. Administration of tocilizumab resulted in a reduction in blood proinflammatory markers such as CRP, improved chest CT findings, restoration of lymphopenia, and improved oxygen support. Subcutaneous administration of tocilizumab ^74^ was also observed to reduce oxygen support requirements in patients with multiple co-morbid conditions. A further clinical trial (NCT04317092) is underway which will provide clear insight into this drug’s therapeutic efficacy in treating COVID-19. Besides using IL-6 as a therapeutic target for COVID-19, an inhibitor against IL-1 has also been subjected to investigation for its effect upon disease progression ^75^. Anakinra is an IL-1α and IL-β receptor blocker which has been observed to abrogate IL-1 signaling and thus been employed as a treatment for multiple autoimmune disorders ^89^. Upon treatment with anakinra for 21 days, significant restoration of serum CRP level was noted in comparison to the control group. Improvement in respiratory function was also associated with significant reduction in mortality with respect to the control group. A similar strategy aiming to inhibit the effects of proinflammatory mediators by the use of the anti-inflammatory drug baricitinib has been shown to successfully control disease progression ^77^. Baricitinib, reported to inhibit Janus kinase activation, is licensed for treating autoimmune conditions such as rheumatoid arthritis ^90^. Treatment of a small group of COVID-19 patients using baricitinib resulted in significant improvement of clinical and respiratory functions along with drastic reduction in time to recovery. Promising results in COVID-19 clinical trials aiming to inhibit proinflammatory mediators thus underscore the role of cytokine storm in disease severity and outcome. A study by Hung *et al.* ^76^ provided the outcome of COVID-19 patients upon use of a drug combination consisting of IFN beta-Ib, lopinavir-ritonavir, and ribavirin. Use of this combination led to rapid clearance of viral RNA from nasopharyngeal and oropharyngeal samples, which was also accompanied by rapid alleviation of symptoms in comparison with the control group.

An unpublished study ^91^ aiming to evaluate the neutralizing potential of antibodies against SARS-CoV-2 in a recovered COVID-19 cohort points to a very dynamic range of neutralizing activity, as exhibited by an undetectable level of the virus in some patients. Albeit this finding raises concerns regarding the potential of antibody response to protect against reinfection by SARS-CoV-2, a report by Chandrashekar *et al*. established a macaque model for SARS-CoV-2 infection wherein re-challenge with SARS-CoV-2 failed to induce reinfection in a humoral as well as cell-mediated immunity-dependent fashion. In agreement with the study mentioned above stating the role of SARS-CoV-2-induced humoral immunity in preventing reinfection, a study by Duan *et al*. ^78^ demonstrated the effectiveness of convalescent plasma from recovered COVID-19 patients in treating individuals suffering from severe COVID-19. Parameters like serum CRP level and lymphocyte count were found to be restored to normal values. Significant improvement was observed in radiological chest findings accompanied by clearance of viral RNA post-plasma therapy. This study thus highlights the low risk of treatment and potential of convalescent plasma therapy in neutralizing viral particles and thus conferring protection against severe COVID-19 progression and outcome. Another study estimating the efficacy of convalescent serum as a treatment in a larger cohort is currently underway (NCT04264858), which upon completion will help direct therapeutic strategies in the face of this pandemic.

Researchers across the world are racing against time to develop a vaccine against 2019-nCoV. However, because of the lack of any existing vaccine against coronaviruses, building the processes and capacities will demand painstaking labor and time. A joint effort by Moderna and the vaccine research center at the National Institutes of Health has led to the design of an mRNA-based vaccine (mRNA for S gene encapsulated in lipid nanoparticles ^92^), which is currently undergoing a recently started phase 1 clinical trial (NCT04283461). Curevac is also working on an mRNA-based vaccine which has been reported very recently to exhibit promising results in preclinical analysis, as denoted by the development of a significant titer of neutralizing SARS-CoV-2-antibodies ^93^. A very recent study by Zhu *et al*. ^79^ assessed the safety profile and efficacy of an adenoviral vector-based RNA vaccine possessing the gene for S protein. This vaccine candidate was found to be well tolerated and capable of eliciting both B- and T-cell immunity 28 and 14 days after administration, respectively, thus warranting further large-scale study before it can be utilized for prophylaxis against SARS-CoV-2 infection. Realistically, considering the time required for efficacy and safety assessment, the establishment of mass production processes, large-scale vaccine production, and distribution in both high- and low-income countries followed by vaccination, which requires trained individuals, it will be very difficult for a SARS-CoV-2 vaccine to be available before another 12–18 months ^94^.

## Conclusion

Infection by SARS-CoV-2 has been declared a global health emergency owing to the rapid spread of the virus irrespective of national boundaries and the destructive impacts it has imposed upon the healthcare system as well as the economy. The development of a vaccine is a time-consuming, cumbersome, and expensive process, especially when novel vaccine platforms are being proposed for disease control. On the other hand, the repurposing/repositioning of existing drugs might provide researchers with a more realistic way to search for and use already approved drugs in this time of urgent need. Although scientists across the world are continuing their search for therapies or vaccines to fight off SARS-CoV-2 infection, a wide gap still exists in our knowledge of SARS-CoV-2 biology. Owing to the lack of any definitive proof of the virus’ origin or regarding the role of any intermediate carrier/reservoir animal species, further work aiming to elucidate the viral emergence and molecular basis of species barrier jump might help in the prevention of any future pandemics like the current one. Further understanding of disease progression warrants the development of an animal model of SARS-CoV-2 infection which might provide detailed insights into the disease’s biology and point towards the development of multiple possible therapeutic strategies.

## References

[1] Hui DSC Wong PC Wang C: SARS: Clinical features and diagnosis. *Respirology.* 2003;8 Suppl(Suppl 1):S20–4. 10.1046/j.1440-1843.2003.00520.x 15018129 PMC7169175

[2] Assiri A Al-Tawfiq JA Al-Rabeeah AA: Epidemiological, demographic, and clinical characteristics of 47 cases of Middle East respiratory syndrome coronavirus disease from Saudi Arabia: A descriptive study. *Lancet Infect Dis.* 2013;13(9):752–61. 10.1016/S1473-3099(13)70204-4 23891402 PMC7185445

[3] Lu R Zhao X Li J: Genomic characterisation and epidemiology of 2019 novel coronavirus: Implications for virus origins and receptor binding. *Lancet.* 2020;395(10224):565–74. 10.1016/S0140-6736(20)30251-8 32007145 PMC7159086

[4] Chan JFW Yuan S Kok KH: A familial cluster of pneumonia associated with the 2019 novel coronavirus indicating person-to-person transmission: A study of a family cluster. *Lancet.* 2020;395(10223):514–23. 10.1016/S0140-6736(20)30154-9 31986261 PMC7159286

[5] Zhang T Wu Q Zhang Z: Probable Pangolin Origin of SARS-CoV-2 Associated with the COVID-19 Outbreak. *Curr Biol.* 2020;30(7):1346–1351.e2. 10.1016/j.cub.2020.03.022 32197085 PMC7156161

[6] Liu P Jiang JZ Wan XF: Are pangolins the intermediate host of the 2019 novel coronavirus (SARS-CoV-2)? *PLoS Pathog.* 2020;16(5):e1008421. 10.1371/journal.ppat.1008421 32407364 PMC7224457

[7] Andersen KG Rambaut A Lipkin WI: The proximal origin of SARS-CoV-2. *Nat Med.* 2020;26(4):450–2. 10.1038/s41591-020-0820-9 32284615 PMC7095063

[8] Li Q Guan X Wu P: Early Transmission Dynamics in Wuhan, China, of Novel Coronavirus-Infected Pneumonia. *N Engl J Med.* 2020;382(13):1199–207. 10.1056/NEJMoa2001316 31995857 PMC7121484

[9] Chapman HA Wei Y Montas G: Reversal of TGF *β*1-Driven Profibrotic State in Patients with Pulmonary Fibrosis. *N Engl J Med.* 2020;382(11):1068–70. 10.1056/NEJMc1915189 32160670 PMC7297220

[10] Holshue ML DeBolt C Lindquist S: First Case of 2019 Novel Coronavirus in the United States. *N Engl J Med.* 2020;382(10):929–36. 10.1056/NEJMoa2001191 32004427 PMC7092802

[11] Sun K Chen J Viboud C: Early epidemiological analysis of the coronavirus disease 2019 outbreak based on crowdsourced data: A population-level observational study. *Lancet Digit Health.* 2020;2(4):e201–e208. 10.1016/S2589-7500(20)30026-1 32309796 PMC7158945

[12] Wu JT Leung K Bushman M: Estimating clinical severity of COVID-19 from the transmission dynamics in Wuhan, China. *Nat Med.* 2020;26(4):506–10. 10.1038/s41591-020-0822-7 32284616 PMC7094929

[13] Delamater PL Street EJ Leslie TF: Complexity of the Basic Reproduction Number (R _0_). *Emerging Infect Dis.* 2019;25(1):1–4. 10.3201/eid2501.171901 30560777 PMC6302597

[14] Song Z Xu Y Bao L: From SARS to MERS, Thrusting Coronaviruses into the Spotlight. *Viruses.* 2019;11(1):59. 10.3390/v11010059 30646565 PMC6357155

[15] Cui J Li F Shi ZL: Origin and evolution of pathogenic coronaviruses. *Nat Rev Microbiol.* 2019;17(3):181–92. 10.1038/s41579-018-0118-9 30531947 PMC7097006

[16] Wu A Peng Y Huang B: Genome Composition and Divergence of the Novel Coronavirus (2019-nCoV) Originating in China. *Cell Host Microbe.* 2020;27(3):325–8. 10.1016/j.chom.2020.02.001 32035028 PMC7154514

[17] Belouzard S Chu VC Whittaker GR: Activation of the SARS coronavirus spike protein via sequential proteolytic cleavage at two distinct sites. *Proc Natl Acad Sci U S A.* 2009;106(14):5871–6. 10.1073/pnas.0809524106 19321428 PMC2660061

[18] Tortorici MA Veesler D: Structural insights into coronavirus entry. *Adv Virus Res.* 2019;105:93–116. 10.1016/bs.aivir.2019.08.002 31522710 PMC7112261

[19] Millet JK Whittaker GR: Host cell entry of Middle East respiratory syndrome coronavirus after two-step, furin-mediated activation of the spike protein. *Proc Natl Acad Sci U S A.* 2014;111(42):15214–9. 10.1073/pnas.1407087111 25288733 PMC4210292

[20] Klenk HD Garten W: Host cell proteases controlling virus pathogenicity. *Trends Microbiol.* 1994;2(2):39–43. 10.1016/0966-842x(94)90123-6 8162439

[21] Bagdonaite I Wandall HH: Global aspects of viral glycosylation. *Glycobiology.* 2018;28(7):443–67. 10.1093/glycob/cwy021 29579213 PMC7108637

[22] Zhou P Yang XL Wang XG: A pneumonia outbreak associated with a new coronavirus of probable bat origin. *Nature.* 2020;579(7798):270–3. 10.1038/s41586-020-2012-7 32015507 PMC7095418

[23] Hoffmann M Kleine-Weber H Schroeder S: SARS-CoV-2 Cell Entry Depends on ACE2 and TMPRSS2 and Is Blocked by a Clinically Proven Protease Inhibitor. *Cell.* 2020;181(2):271–280.e8. 10.1016/j.cell.2020.02.052 32142651 PMC7102627

[24] Huang C Wang Y Li X: Clinical features of patients infected with 2019 novel coronavirus in Wuhan, China. *Lancet.* 2020;395(10223):497–506. 10.1016/S0140-6736(20)30183-5 31986264 PMC7159299

[25] Antonio GE Wong KT Hui DSC: Imaging of severe acute respiratory syndrome in Hong Kong. *AJR Am J Roentgenol.* 2003;181(1):11–7. 10.2214/ajr.181.1.1810011 12818822

[26] Wong CK Lam CW Wu AK: Plasma inflammatory cytokines and chemokines in severe acute respiratory syndrome. *Clin Exp Immunol.* 2004;136(1):95–103. 10.1111/j.1365-2249.2004.02415.x 15030519 PMC1808997

[27] Chen N Zhou M Dong X: Epidemiological and clinical characteristics of 99 cases of 2019 novel coronavirus pneumonia in Wuhan, China: A descriptive study. *Lancet.* 2020;395(10223):507–13. 10.1016/S0140-6736(20)30211-7 32007143 PMC7135076

[28] Bangash MN Patel J Parekh D: COVID-19 and the liver: Little cause for concern. *Lancet Gastroenterol Hepatol.* 2020;5(6):529–30. 10.1016/S2468-1253(20)30084-4 32203680 PMC7270582

[29] Shi H Han X Jiang N: Radiological findings from 81 patients with COVID-19 pneumonia in Wuhan, China: A descriptive study. *Lancet Infect Dis.* 2020;20(4):425–34. 10.1016/S1473-3099(20)30086-4 32105637 PMC7159053

[30] Imaging M: Original Report. *Rev Lit Arts Am.* 2005;200–4.

[31] Wong KT Antonio GE Hui DSC: Thin-section CT of severe acute respiratory syndrome: Evaluation of 73 patients exposed to or with the disease. *Radiology.* 2003;228(2):395–400. 10.1148/radiol.2283030541 12738877

[32] Das KM Lee EY Enani MA: CT correlation with outcomes in 15 patients with acute Middle East respiratory syndrome coronavirus. *AJR Am J Roentgenol.* 2015;204(4):736–42. 10.2214/AJR.14.13671 25615627

[33] Ajlan AM Ahyad RA Jamjoom LG: Middle East respiratory syndrome coronavirus (MERS-CoV) infection: Chest CT findings. *AJR Am J Roentgenol.* 2014;203(4):782–7. 10.2214/AJR.14.13021 24918624

[34] Inciardi RM Lupi L Zaccone G: Cardiac Involvement in a Patient With Coronavirus Disease 2019 (COVID-19). *JAMA Cardiol.* 2020. 10.1001/jamacardio.2020.1096 32219357 PMC7364333

[35] Van den Berghe G Bouillon R Mesotten D: Glucose control in critically ill patients. *N Engl J Med.* 2009;361(1):89; author reply91–2. 10.1056/NEJMc090812 19571290

[36] Helms J Kremer S Merdji H: Neurologic Features in Severe SARS-CoV-2 Infection. *N Engl J Med.* 2020;382(23):2268–2270. 10.1056/NEJMc2008597 32294339 PMC7179967

[37] Mao L Jin H Wang M: Neurologic Manifestations of Hospitalized Patients With Coronavirus Disease 2019 in Wuhan, China. *JAMA Neurol.* 2020;77(6):1–9. 10.1001/jamaneurol.2020.1127 32275288 PMC7149362

[38] Bénézit F Le Turnier P Declerck C: Utility of hyposmia and hypogeusia for the diagnosis of COVID-19. *Lancet Infect Dis.* 2020;S1473-3099(20)30297-8. 10.1016/S1473-3099(20)30297-8 32304632 PMC7159866

[39] Moriguchi T Harii N Goto J: A first case of meningitis/encephalitis associated with SARS-Coronavirus-2. *Int J Infect Dis.* 2020;94:55–8. 10.1016/j.ijid.2020.03.062 32251791 PMC7195378

[40] Yang X Yu Y Xu J: Clinical course and outcomes of critically ill patients with SARS-CoV-2 pneumonia in Wuhan, China: A single-centered, retrospective, observational study. *Lancet Respir Med.* 2020;8(5):475–81. 10.1016/S2213-2600(20)30079-5 32105632 PMC7102538

[41] Bhat TA Kalathil SG Bogner PN: An Animal Model of Inhaled Vitamin E Acetate and EVALI-like Lung Injury. *N Engl J Med.* 2020;382(12):1175–7. 10.1056/NEJMc2000231 32101656 PMC7299285

[42] Tsang TK Cowling BJ Fang VJ: Influenza A Virus Shedding and Infectivity in Households. *J Infect Dis.* 2015;212(9):1420–8. 10.1093/infdis/jiv225 25883385 PMC4601913

[43] Peiris JSM Chu CM Cheng VCC: Clinical progression and viral load in a community outbreak of coronavirus-associated SARS pneumonia: A prospective study. *Lancet.* 2003;361(9371):1767–72. 10.1016/s0140-6736(03)13412-5 12781535 PMC7112410

[44] Zhao J Zhao J Legge K: Age-related increases in PGD _2_ expression impair respiratory DC migration, resulting in diminished T cell responses upon respiratory virus infection in mice. *J Clin Invest.* 2011;121(12):4921–30. 10.1172/JCI59777 22105170 PMC3226008

[45] Koven S: They Call Us and We Go. *N Engl J Med.* 2020;382(21):1978–9. 10.1056/NEJMp2009027 32283001

[46] Molinaro GA Lee DA Parashar UD: Rotavirus vaccines. *N Engl J Med.* 2006;354(16):1747-51; author reply1747-51. 10.1056/NEJMc060253 16625014

[47] January F Hospital T Hospital T: Correspondence Detection of Covid-19 in Children in Early January 2020 in Wuhan, China.2020.

[48] Xu Y Li X Zhu B: Characteristics of pediatric SARS-CoV-2 infection and potential evidence for persistent fecal viral shedding. *Nat Med.* 2020;26(4):502–505. 10.1038/s41591-020-0817-4 32284613 PMC7095102

[49] Qiu H Wu J Hong L: Clinical and epidemiological features of 36 children with coronavirus disease 2019 (COVID-19) in Zhejiang, China: An observational cohort study. *Lancet Infect Dis.* 2020;20(6):689–696. 10.1016/S1473-3099(20)30198-5 32220650 PMC7158906

[50] Ct C: Comment Novel paediatric presentation of COVID-19 with ARDS and cytokine storm syndrome without respiratory symptoms.2020;2(20):19–21. 10.1016/S2665-9913(20)30137-5 PMC7228732 32427161

[51] Dallan C Romano F Siebert J: Septic shock presentation in adolescents with COVID-19. *Lancet Child Adolesc Health.* 2020;4(7):e21–e23. 10.1016/S2352-4642(20)30164-4 32442421 PMC7237371

[52] Cook J Harman K Zoica B: Horizontal transmission of severe acute respiratory syndrome coronavirus 2 to a premature infant: Multiple organ injury and association with markers of inflammation. *Lancet Child Adolesc Health.* 2020;4(7):548–551 10.1016/S2352-4642(20)30166-8 32442422 PMC7237364

[53] Chen H Guo J Wang C: Clinical characteristics and intrauterine vertical transmission potential of COVID-19 infection in nine pregnant women: A retrospective review of medical records. *The Lancet.* 2020;395(10226):809–15. 10.1016/S0140-6736(20)30360-3 32151335 PMC7159281

[54] Yu N Li W Kang Q: Clinical features and obstetric and neonatal outcomes of pregnant patients with COVID-19 in Wuhan, China: A retrospective, single-centre, descriptive study. *Lancet Infect Dis.* 2020;20(5):559–64. 10.1016/S1473-3099(20)30176-6 32220284 PMC7158904

[55] Wichmann D Sperhake JP Lütgehetmann M: Autopsy Findings and Venous Thromboembolism in Patients With COVID-19. *Ann Intern Med.* 2020;M20-2003. 10.7326/M20-2003 33316197

[56] Menter T Haslbauer JD Nienhold R: Post-mortem examination of COVID19 patients reveals diffuse alveolar damage with severe capillary congestion and variegated findings of lungs and other organs suggesting vascular dysfunction. *Histopathology.* 2020. 10.1111/his.14134 32364264 PMC7496150

[57] Fox SE Akmatbekov A Harbert JL: Pulmonary and Cardiac Pathology in Covid-19: The First Autopsy Series from New Orleans. *medRxiv.* 2020 10.1101/2020.04.06.20050575 PMC7255143 32473124

[58] Varga Z Flammer AJ Steiger P: Endothelial cell infection and endotheliitis in COVID-19. *Lancet.* 2020;395(10234):1417–8. 10.1016/S0140-6736(20)30937-5 32325026 PMC7172722

[59] Ziegler CGK Allon SJ Nyquist SK: SARS-CoV-2 Receptor ACE2 Is an Interferon-Stimulated Gene in Human Airway Epithelial Cells and Is Detected in Specific Cell Subsets across Tissues. *Cell.* 2020;181(5):1016–1035.e19. 10.1016/j.cell.2020.04.035 32413319 PMC7252096

[60] Liao M Liu Y Yuan J: Single-cell landscape of bronchoalveolar immune cells in patients with COVID-19. *Nat Med.* 2020;26(6):842–844. 10.1038/s41591-020-0901-9 32398875

[61] Tay MZ Poh CM Rénia L: The trinity of COVID-19: Immunity, inflammation and intervention. *Nat Rev Immunol.* 2020;20(6):363–374. 10.1038/s41577-020-0311-8 32346093 PMC7187672

[62] Blanco-Melo D Nilsson-Payant BE Liu WC: Imbalanced Host Response to SARS-CoV-2 Drives Development of COVID-19. *Cell.* 2020;181(5):1036–1045.e9. 10.1016/j.cell.2020.04.026 32416070 PMC7227586

[63] Cao W Li T: COVID-19: Towards understanding of pathogenesis. *Cell Res.* 2020;30(5):367–9. 10.1038/s41422-020-0327-4 32346073 PMC7186532

[64] Zheng M Gao Y Wang G: Functional exhaustion of antiviral lymphocytes in COVID-19 patients. *Cell Mol Immunol.* 2020;17(5):533–5. 10.1038/s41423-020-0402-2 32203188 PMC7091858

[65] Liu Y Du X Chen J: Neutrophil-to-lymphocyte ratio as an independent risk factor for mortality in hospitalized patients with COVID-19. * J Infect.* 2020;81(1):e6–e12. 10.1016/j.jinf.2020.04.002 32283162 PMC7195072

[66] Zuo Y Yalavarthi S Shi H: Neutrophil extracellular traps in COVID-19. *JCI Insight.* 2020;5(11):138999. 10.1172/jci.insight.138999 32329756 PMC7308057

[67] Sawalha AH Zhao M Coit P: Epigenetic dysregulation of ACE2 and interferon-regulated genes might suggest increased COVID-19 susceptibility and severity in lupus patients. *Clin Immunol.* 2020;215:108410. 10.1016/j.clim.2020.108410 32276140 PMC7139239

[68] McGonagle D O'Donnell JS Sharif K: Immune mechanisms of pulmonary intravascular coagulopathy in COVID-19 pneumonia. *Lancet Rheumatol.* 2020 10.1016/S2665-9913(20)30121-1 PMC7252093 32835247

[69] Wang Y Zhang D Du G: Remdesivir in adults with severe COVID-19: A randomised, double-blind, placebo-controlled, multicentre trial. *Lancet.* 2020;395(10236):1569–78. 10.1016/S0140-6736(20)31022-9 32423584 PMC7190303

[70] Tang W Cao Z Han M: Hydroxychloroquine in patients with mainly mild to moderate coronavirus disease 2019: Open label, randomised controlled trial. *BMJ.* 2020;369:m1849. 10.1136/bmj.m1849 32409561 PMC7221473

[71] Cao B Wang Y Wen D: A Trial of Lopinavir-Ritonavir in Adults Hospitalized with Severe Covid-19. *N Engl J Med.* 2020;382(19):1787–99. 10.1056/NEJMoa2001282 32187464 PMC7121492

[72] Cai Q Yang M Liu D: Experimental Treatment with Favipiravir for COVID-19: An Open-Label Control Study. *Engineering (Beijing).* 2020. 10.1016/j.eng.2020.03.007 32346491 PMC7185795

[73] Xu X Han M Li T: Effective treatment of severe COVID-19 patients with tocilizumab. *Proc Natl Acad Sci U S A.* 2020;117(20):10970–5. 10.1073/pnas.2005615117 32350134 PMC7245089

[74] Mazzitelli M Arrighi E Serapide F: Use of subcutaneous tocilizumab in patients with COVID-19 pneumonia. *J Med Virol.* 2020. 10.1002/jmv.26016 32410234 PMC7272953

[75] Cavalli G de Luca G Campochiaro C: Interleukin-1 blockade with high-dose anakinra in patients with COVID-19, acute respiratory distress syndrome, and hyperinflammation: A retrospective cohort study. *Lancet Rheumatol.* 2020;2(6):e325–e331. 10.1016/S2665-9913(20)30127-2 32501454 PMC7252085

[76] Hung IFN Lung KC Tso EYK: Triple combination of interferon beta-1b, lopinavir–ritonavir, and ribavirin in the treatment of patients admitted to hospital with COVID-19: An open-label, randomised, phase 2 trial. *Lancet.* 2020;395(10238):1695–1704. 10.1016/S0140-6736(20)31042-4 32401715 PMC7211500

[77] Cantini F Niccoli L Matarrese D: Baricitinib therapy in COVID-19: A pilot study on safety and clinical impact. *J Infect.* 2020;S0163-4453(20)30228-0. 10.1016/j.jinf.2020.04.017 32333918 PMC7177073

[78] Duan K Liu B Li C: Effectiveness of convalescent plasma therapy in severe COVID-19 patients. *Proc Natl Acad Sci U S A.* 2020;117(17):9490–6. 10.1073/pnas.2004168117 32253318 PMC7196837

[79] Zhu FC Li YH Guan XH: Safety, tolerability, and immunogenicity of a recombinant adenovirus type-5 vectored COVID-19 vaccine: A dose-escalation, open-label, non-randomised, first-in-human trial. *Lancet.* 2020;395(10240):1845–1854. 10.1016/S0140-6736(20)31208-3 32450106 PMC7255193

[80] Zhou Y Hou Y Shen J: Network-based drug repurposing for novel coronavirus 2019-nCoV/SARS-CoV-2. *Cell Discov.* 2020;6:14. 10.1038/s41421-020-0153-3 32194980 PMC7073332

[81] Wang M Cao R Zhang L: Remdesivir and chloroquine effectively inhibit the recently emerged novel coronavirus (2019-nCoV) in vitro. *Cell Res.* 2020;30(3):269–71. 10.1038/s41422-020-0282-0 32020029 PMC7054408

[82] Agostini ML Andres EL Sims AC: Coronavirus Susceptibility to the Antiviral Remdesivir (GS-5734) Is Mediated by the Viral Polymerase and the Proofreading Exoribonuclease. *mBio.* 2018;9(2):1953. 10.1128/mBio.00221-18 29511076 PMC5844999

[83] Savarino A Di Trani L Donatelli I: New insights into the antiviral effects of chloroquine. *Lancet Infect Dis.* 2006;6(2):67–9. 10.1016/S1473-3099(06)70361-9 16439323 PMC7129107

[84] Vincent MJ Bergeron E Benjannet S: Chloroquine is a potent inhibitor of SARS coronavirus infection and spread. *Virol J.* 2005;2:69. 10.1186/1743-422X-2-69 16115318 PMC1232869

[85] Liu J Cao R Xu M: Hydroxychloroquine, a less toxic derivative of chloroquine, is effective in inhibiting SARS-CoV-2 infection *in vitro*. *Cell Discov.* 2020;6:16. 10.1038/s41421-020-0156-0 32194981 PMC7078228

[86] Gautret P Lagier JC Parola P: Hydroxychloroquine and azithromycin as a treatment of COVID-19: Results of an open-label non-randomized clinical trial. *Int J Antimicrob Agents.* 2020;105949. 10.1016/j.ijantimicag.2020.105949 32205204 PMC7102549

[87] Chu CM Cheng VCC Hung IFN: Role of lopinavir/ritonavir in the treatment of SARS: Initial virological and clinical findings. *Thorax.* 2004;59(3):252–6. 10.1136/thorax.2003.012658 14985565 PMC1746980

[88] Du YX Chen XP: Favipiravir: Pharmacokinetics and Concerns About Clinical Trials for 2019-nCoV Infection. *Clin Pharmacol Ther.* 2020. 10.1002/cpt.1844 32246834

[89] Cavalli G Dinarello CA: Treating rheumatological diseases and co-morbidities with interleukin-1 blocking therapies. *Rheumatology (Oxford).* 2015;54(12):2134–44. 10.1093/rheumatology/kev269 26209330 PMC5009422

[90] Bechman K Subesinghe S Norton S: A systematic review and meta-analysis of infection risk with small molecule JAK inhibitors in rheumatoid arthritis. *Rheumatology (Oxford).* 2019;58(10):1755–66. 10.1093/rheumatology/kez087 30982883

[91] Xun J Lu L Jiang S: Patient Cohort and Their Implications. *medRxiv.* 2020.

[92] Thanh Le T Andreadakis Z Kumar A: The COVID-19 vaccine development landscape. *Nat Rev Drug Discov.* 2020;19(5):305–6. 10.1038/d41573-020-00073-5 32273591

[93] Coronavirus R Ag C Company T: CureVac ´ s Optimized mRNA Platform Provides Positive Pre-Clinical Results at Low Dose for Coronavirus Vaccine Candidate. 2020 Reference Source

[94] Amanat F Krammer F: SARS-CoV-2 Vaccines: Status Report. *Immunity.* 2020;52(4):583–9. 10.1016/j.immuni.2020.03.007 32259480 PMC7136867

